# The Effects of NF-kB Inhibition with p65-TMD-Linked PTD on Inflammatory Responses at Peri-implantitis Sites

**DOI:** 10.1007/s10753-021-01500-4

**Published:** 2021-06-25

**Authors:** Hyun Jung Jung, Won Lee, Jin-Su Shin, Sang-Kyou Lee, Jae Hoon Lee

**Affiliations:** 1 Department of Prosthodontics, College of Dentistry, Yonsei University, 50-1 Yonsei-ro, Seodaemoon-gu, Seoul, 120-752 South Korea; 2 The Catholic University of Korea, Uijeongbu St. Mary’s Hospital, Uijeongbu, South Korea; 3 Department of Biotechnology, College of Life Science and Biotechnology, Yonsei University, Seoul, South Korea

**Keywords:** NF-kB, nt-p65-TMD, peri-implantitis, inflammation, bone resorption

## Abstract

The objective of this study was to find out if suppression of NF-kB complex function by p65-TMD-linked PTD could reduce host inflammation and bone resorption at peri-implantitis sites in rats. Twenty-one male 5-week-old SD rats were divided into three groups: untreated control group (A), silk-induced peri-implantitis group (B), and nt (nucleus transducible)-p65-TMD-treated, silk-induced peri-implantitis group (C). Implant sulcus of a rat in group C were divided into two groups, namely group Cp and Cb. Palatal implant sulcus where nt-p65-TMD solution was applied with an insulin syringe were assigned to group Cp. Buccal implant sulcus without topical nt-p65-TMD application were assigned to group Cb. H&E staining, TRAP staining, and immunohistological staining were done. The crestal bone levels of group A were significantly higher than those of group B at p<0.01. The crestal bone levels of group Cp were significantly higher than those of group Cb at p<0.05. H-E staining showed increased apical migration of junctional epithelium and inflammatory cells in group Cb. TRAP staining revealed more multinucleated osteoclasts in group Cb. As for immunohistological staining, group Cb showed many IL-6-positive cells while group Cp had none. In this study, p65-TMD-linked PTD inhibited NF-kB functions and reduced inflammation and bone resorption at peri-implantitis sites in rats.

## INTRODUCTION

Dental implant installment has become the gold standard and routine procedure for rehabilitating edentulous patients. As the number of patients benefitting from implant treatment has increased, so has the number of related complications, including peri-implantitis (a cross-sectional study by Fransson *et al.* reported peri-implantitis in 28% of implants installed for 5–20 years) [1]. Bacteria are widely recognized as the cause of periodontitis, including peri-implantitis, and inflammation plays an important role in its progress. Thus, controlling host inflammatory response can reduce major complications of peri-implantitis such as periodontal bone loss [2]. Lipid-based mediators such as prostaglandins, leukotrienes, other cytokines, and chemokines activate bone loss in a host. Cytokines in particular play a major role in bacteria-induced periodontal bone loss. In an experiment on mice, inhibition of interleukin-6 (IL-6) reduced bone resorption [3].

In this study, NF-kB complex, a transcription factor activated by bacteria-induced and inflammatory cytokines, was competitively inhibited to reduce host inflammatory response, particularly bone loss in peri-implantitis-induced rats [4]. Rats were chosen as experimental subjects because they are human homologs, easy to induce peri-implantitis, well-known experimental models for peri-implantitis, and much more cost-effective than beagles, which are also popular subjects in preclinical animal studies on periodontitis and peri-implantitis. In comparison with other experiments which used non-steroidal anti-inflammatory drugs (NSAID) to inhibit inflammation, inhibition of NF-kB nuclear factor has the advantage of lower cellular toxicity and thus fewer side effects due to selective inhibition of inflammatory signaling pathways. NSAID inhibits the cyclooxygenase pathway of arachidonic acid metabolism and reduces tissue damage by periodontitis. However, based on clinical studies, long-term inhibition of cyclooxygenase is not recommended due to side effects such as gastrointestinal erosions, as well as renal and hepatic insufficiency [5].

In order to inhibit NF-kB complex, this study used p65-transcription modulation domain (TMD)–linked protein transduction domain (PTD). A previous study created p65-TMD-PTD from a mouse, in which TMD competed with NF-kB complex in binding DNA and did not induce transcription [6]. The study also reported that cell viability of the p65-TMD-PTD-treated group was similar to that of the control group [6]. As in the previous study, PTD was used to transduce p65-TMD in this experiment. PTD, a small peptide composed of 10 to 16 basic amino acids, passes through plasma membranes with linked materials, such as various proteins, nucleic acid, and nanoparticles, and shows a tendency to accumulate inside cells and transport into nuclei [7]. This protein-based strategy maximizes the efficiency of delivery because of its low toxicity and high transduction efficiency [7]. Also, the use of PTD allows local delivery, which can target the treatment area and thus cause fewer side effects in comparison with systemic delivery. Park *et al.* reported successful transduction of nt-p65-TMD into nuclei and subsequent inhibition of DNA transcription. In addition, Oh *et al.* used the same PTD as Park *et al.* to transfer hypoxia-inducible factor 1α (HIF-1α), a transcription factor whose function of angiogenesis and osteogenesis are normally inhibited under hyperglycemic conditions in diabetic patients [8]. Exogenous HIF-1α was administered via PTD, resulting in successful upregulation of gene expression more favorable to bone formation in diabetic mice. Due to the successful results in previous similar studies, PTD seemed a viable option for transporting protein in this experiment.

In this study, NF-kB complex was competitively inhibited using p65-TMD-PTD to reduce host inflammation and bone resorption around peri-implantitis sites in rats. Since the NF-kB complex pathway plays a crucial role in inflammation, it is highly probable that successful local inhibition of the NF-kB complex pathway may reduce inflammatory response and crestal bone resorption in peri-implantitis.

## MATERIALS AND METHODS

### Generation and Purification of the Nucleus-Transducible Form of nt-p65-TMD

The FLAG-tagged p65-DBD (p65-TMD) that encodes amino acids (1–187) conjugated with Hph-1-PTD from the full-length mouse p65 (1–551) was PCR-amplified. The same protein was used in Park *et al.* [6]. The intracellular transduction efficiency of nt-p65-TMD was tested by treating BV2 and Jurkat T cells with nt-p65-TMD and confirming its presence after 48 h by SDS-PAGE [6]. Also, the functional efficacy of nt-p65-TMD was tested by co-transfecting HEK293T cells with the vectors expressing wild-type p65 and NFKB1-driven luciferase promoter. When the transfected cells were treated with nt-p65-TMD, the luciferase activities were significantly inhibited while non-transducible p65-TMD could not affect these activities [6]. In this experiment, nt-p65-TMD solution was purified to a concentration of 1.2μg/μl, the highest concentration possible after purification. A detailed description of the protein generation can be found in section 2.1 of “Intranuclear interactomic inhibition of NF-kB suppresses LPS-induced severe sepsis” by Park *et al.* [6].

### Implants

**Fig. 1:**
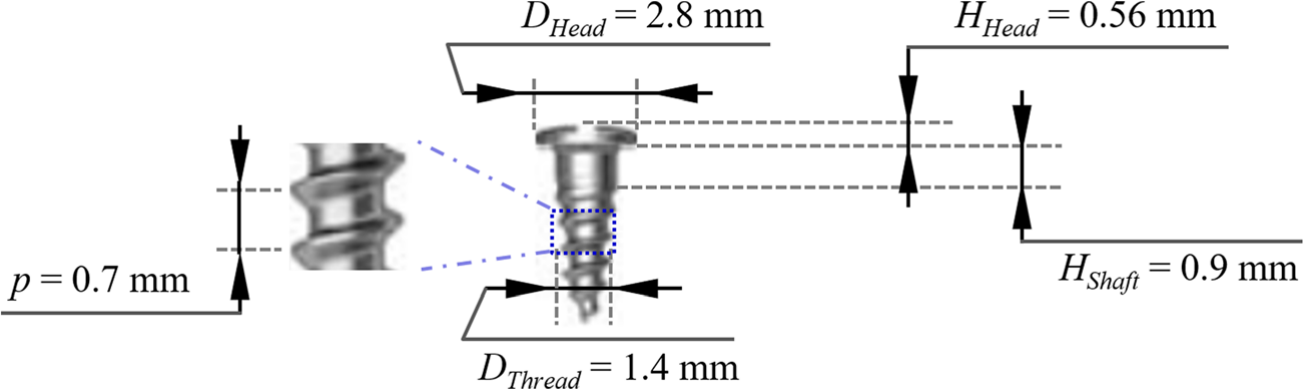
Diagram of the bone screw: Osstem BSCH 1404.

Bone screw implants (BSCH1404, Osstem, Seoul, Korea) 1.4 mm in diameter and 4 mm in length were used in this study. The detailed diagram of the bone screw with dimensions is depicted in Fig. 1. They are made of Ti-6Al-4V, machine-surfaced, and sterilized. The distance from the implant top to the first thread is 1.46 mm.

### Experimental Animals

Four-week-old male SD rats were ordered from Orientbio (Gapyeong-gun, South Korea). Four-week-old rats initially weighed about 100–120g, and about 150–200g after 1-week acclimation. Environmental conditions were a temperature of 21°C ± 2°C, humidity of 45~55%, and a 12:12 light: dark cycle. Two rats each were housed in 260x420x180 mm cages (Jeung Do Bio & Plant Co., Ltd., Seoul, South Korea) with Lignocel® hygienic animal bedding (Rosenberg, Germany) and given free access to breeding diet (Altromin 1314 IRR, North Rhine-Westphalia, Germany) and water. Their health status was monitored twice daily. All rats were maintained in specific pathogen-free conditions in the Clinical Research Laboratory, Uijeongbu St. Mary’s Hospital, Catholic University. Animal care and experimental procedures were performed in accordance with the Guidelines for Animal Experimentation of Catholic University and with the approval of the Institutional Animal Care and Use Committee (UJA2017-02A15A).

### Experimental Design

**Fig. 2:**
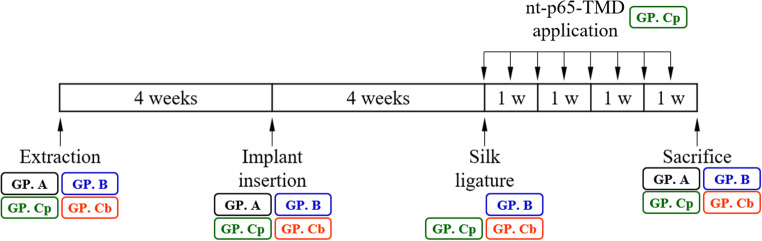
Experimental procedure: timetable (GP. A: untreated control group, GP. B: silk-induced peri-implantitis group, GP. Cp: nt-p65-TMD-treated palatal implant sulcus group, GP. Cb: buccal implant sulcus without nt-p65-TMD treatment group).

Twenty-one male 5-week-old SD rats (Orientbio, Gapyeong-gun, South Korea) were used in this study. G power analysis (G power 3.1.9.2) was used to calculate the total number size of 21 when alpha is 0.05, power 0.95, effect size 0.98, and predictor 3. The maxillary left first molars were extracted under general anesthesia with an intraperitoneal injection of 60~100 mg/kg ketamine (Yuhan, Seoul, South Korea) and10 mg/kg rompun (Bayer Korea, Ansan, South Korea). After extraction, 0.05 mg/kg ketoprofen (Ubtech, Anyang, South Korea) and 4 mg/kg gentamicin (Samu Median Co., Yesan-gun, South Korea) were injected as an analgesic and antibiotic, respectively. Extraction sockets were healed after 4 weeks and prepared for implant insertion. Following general anesthesia using the same procedure 4 weeks after extraction, the recipient sites for implantation were drilled with a low-speed dental handpiece equipped with an implant drill (diameter 1.2 mm). Sterilized saline irrigation was maintained throughout the process. Bone screw implants were inserted down to the first thread using a driver. Ten Ncm torque was applied for implant placement. Ketoprofen and gentamicin were administered after the surgery. The rats were divided into three groups: untreated control group (A), silk-induced peri-implantitis group (B), and nt-p65-TMD-treated, silk-induced peri-implantitis group (C). Implant sulcus of a rat in group C were divided into two groups, namely group Cp and Cb. Palatal implant sulcus where nt-p65-TMD solution was applied with an insulin syringe were assigned to group Cp. Buccal implant sulcus without topical nt-p65-TMD application were assigned to group Cb. Seven rats were randomly assigned to each group using the standard = RAND() function in Microsoft Excel. Implant mobility was tested by tactile perception to check osseointegration 4 weeks after implant insertion. Rats with mobile implants were considered failure and excluded. Rats in group A were untreated. Under inhalation anesthesia using 1.34% isoflurane (Aesica Queenborough Limited, Queenborough, UK), 5–0 silk was tied around implants in groups B and C 4 weeks post-implantation [9]. After silk ligatures were tied, rats from group Cp received topical application of 25-μl nt-p65-TMD solution (1.2 μg/μl) in the palatal implant sulcus with an insulin syringe under inhalation anesthesia twice every week for 4 weeks. Inhalation anesthesia was used for non-invasive procedures because it was far less stressful to animals and thus could be used frequently for nt-p65-TMD application. All rats from the three groups were injected with ketamine and sacrificed using carbon dioxide gas 8 weeks after implant insertion (Fig. 2). All experimental procedures were carried out in the morning except nt-p65-TMD application, which had to be done during lunch time due to its frequency.

### Preparation of Specimens

The left maxillary region, containing the left second molar as an orientation reference to distinguish between the buccal and palatal sides of the implants, was resected from each rat and fixed in 4% paraformaldehyde/PBS at 4°C for 10h. The specimens were decalcified by immersion in 10% ethylenediamine tetraacetic acid (EDTA)·2Na (pH 7.4) at 35°C for 16 weeks, using a PelcoBioWave®Pro tissue processor microwave system (CA, USA). After demineralization, the implants in the left maxillary region were easily removed from decalcified specimens with a pincette. The specimens were then dehydrated in an ascending ethanol series and embedded in paraffin using the Sakura Tissue-Tek®VIP^TM^ tissue embedding console system (MN, USA). Buccopalatal serial sections (4-μm thickness) were cut and collected using a Leica RM2255 (Nussloch, Germany).

### Histopathological Study

Sections obtained from the specimens were stained with hematoxylin and eosin for histopathological observation following the H&E Harris staining protocol. The sections from groups A and B were used to measure the distance from the alveolar bone crest to the implant bottom (the total embedded distance) to compare results and confirm that silk ligatures induced inflammation and bone resorption. The sections from group C were used to measure differences in the distance between palatal and buccal alveolar bone crests. CellSens software (Tokyo, Japan) was used to make all measurements at magnifications of ×15 and ×40.

In addition, tartrate-resistant acid phosphatase (TRAP) staining using #MK300 Takara (Kusatsu, Japan) was done to observe osteoclast distribution in crestal bone around the implants.

### Immunohistological Staining

Anti-IL-6 antibody (bs-0782R, Bioss, Woburn, MA, USA) was used for immunohistological staining of the prepared sections to detect IL-6 expression. The concentration/dilution of the antibodies was 1:100. After the process of deparaffinization, hydration, and washing, sections were immersed overnight in rabbit polyclonal anti-IL-6 antibody. They were then incubated with goat anti-rabbit secondary antibodies for 10 min. The treated sections were counterstained with hematoxylin.

### Statistical Analysis

All statistical analyses were done using Microsoft Excel. Differences between groups A and B were evaluated using the Mann-Whitney U-test. Level of statistical significance was set at p<0.05. Differences between palatal and buccal crestal bones in groups Cp and Cb were compared using the same test with the p-value set at 0.05.

## RESULTS

### Histopathological Findings

**Fig. 3:**
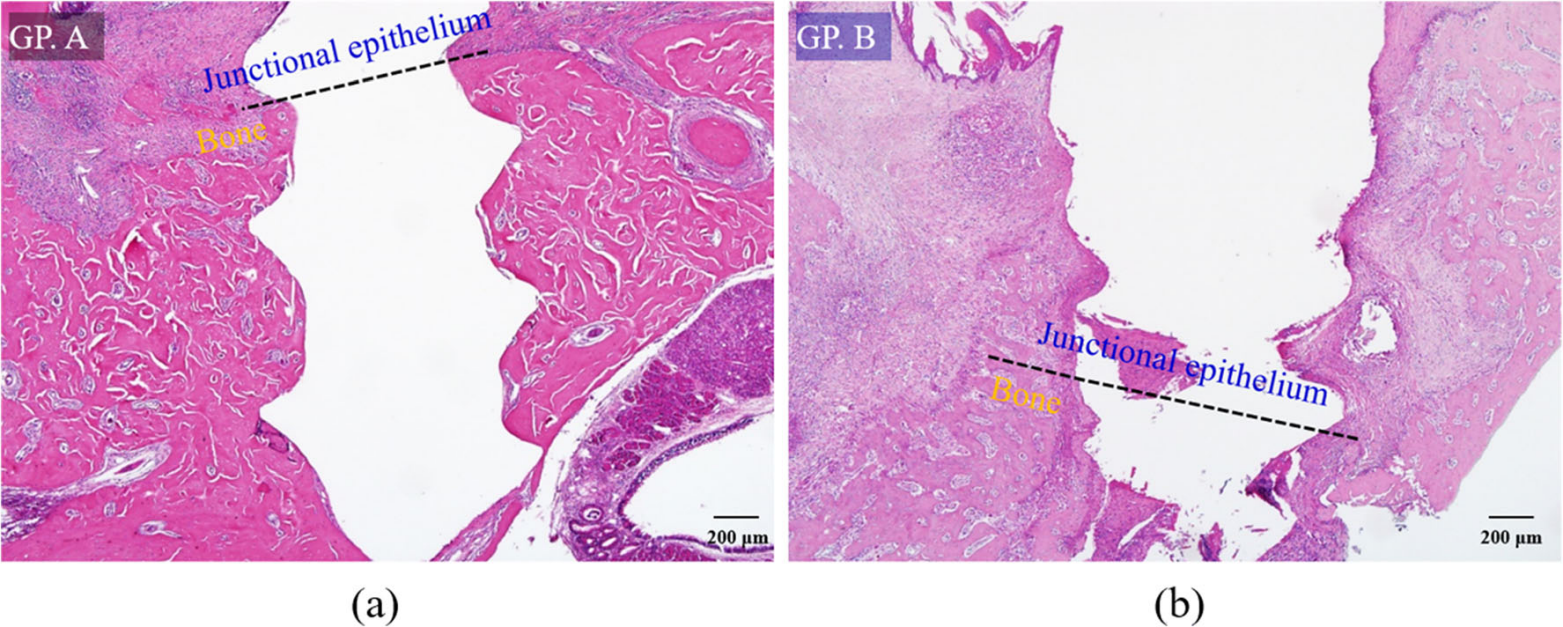
H-E-stained specimen slide: (a) untreated control group A (magnification ×40) and (b) silk-induced peri-implantitis group B (×40). Group A showed the highest crestal bone level and least apical migration of junctional epithelium while group B had the most crestal bone resorption and apical migration of junctional epithelium.

H-E-stained slides of the control group A showed the highest crestal bone level, least apical migration of junctional epithelium, and no osteoclasts on alveolar bone surface while those of group B had the most apical migration of junctional epithelium and crestal bone resorption, accompanied by many osteoclasts (Fig. 3). Also, inflammatory cell infiltration was observed in the junctional epithelium and adjacent connective tissue of group B. These results verified that the silk ligature around the bone screw induced inflammation and bone resorption.

**Fig. 4:**
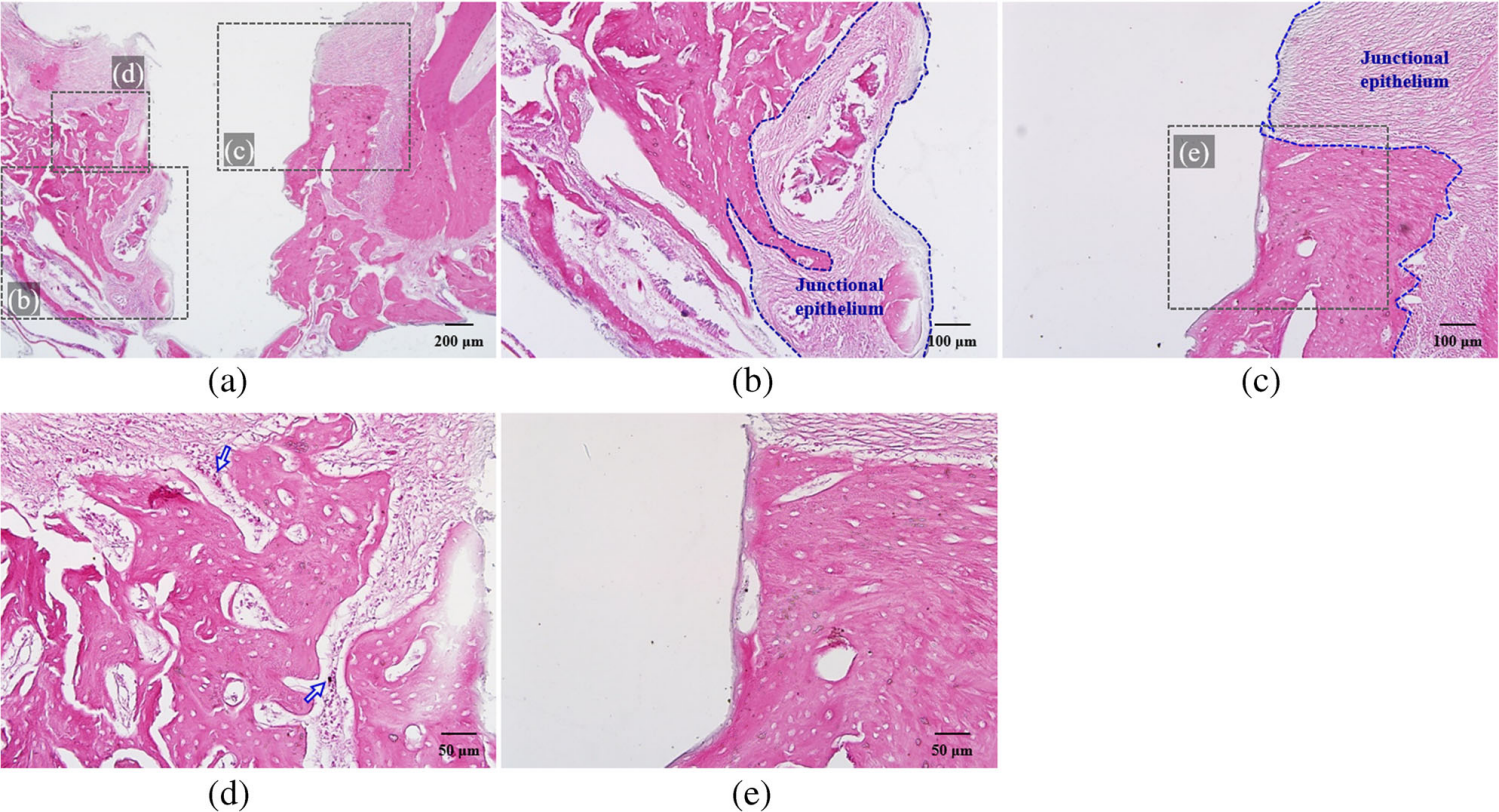
H-E-stained specimen slide: (a) nt-p65-TMD-treated and silk-induced peri-implantitis group C (magnification ×40), (b) group Cb, which had buccal implant sulcus without topical nt-p65-TMD application (×100), and (c) group Cp, which had palatal implant sulcus with topical nt-p65-TMD application (×100). Group Cb showed more apical migration of junctional epithelium, infiltration of inflammatory cells, and bone resorption than group Cp. (d) In group Cb (×200), one of the lymphocytes with clocklike nuclei and osteoclasts, stained as dark spots, was highlighted with arrows. (e) group Cb (×200) showed few inflammatory cells and osteoclasts.

H-E-stained slides showed increased apical migration of junctional epithelium and infiltration of inflammatory cells in the peri-implant junctional epithelium and connective tissue of group Cb. More bone resorption accompanied by osteoclasts was also observed (Fig 4. b). On the contrary, reduced apical migration of junctional epithelium and less bone resorption with fewer inflammatory cells and osteoclasts were detected in group Cp (Fig. 4 c, e). One of the lymphocytes with clockface nuclei and osteoclasts, stained as dark spots, was highlighted with arrows (Fig. 4 d).

**Fig. 5:**
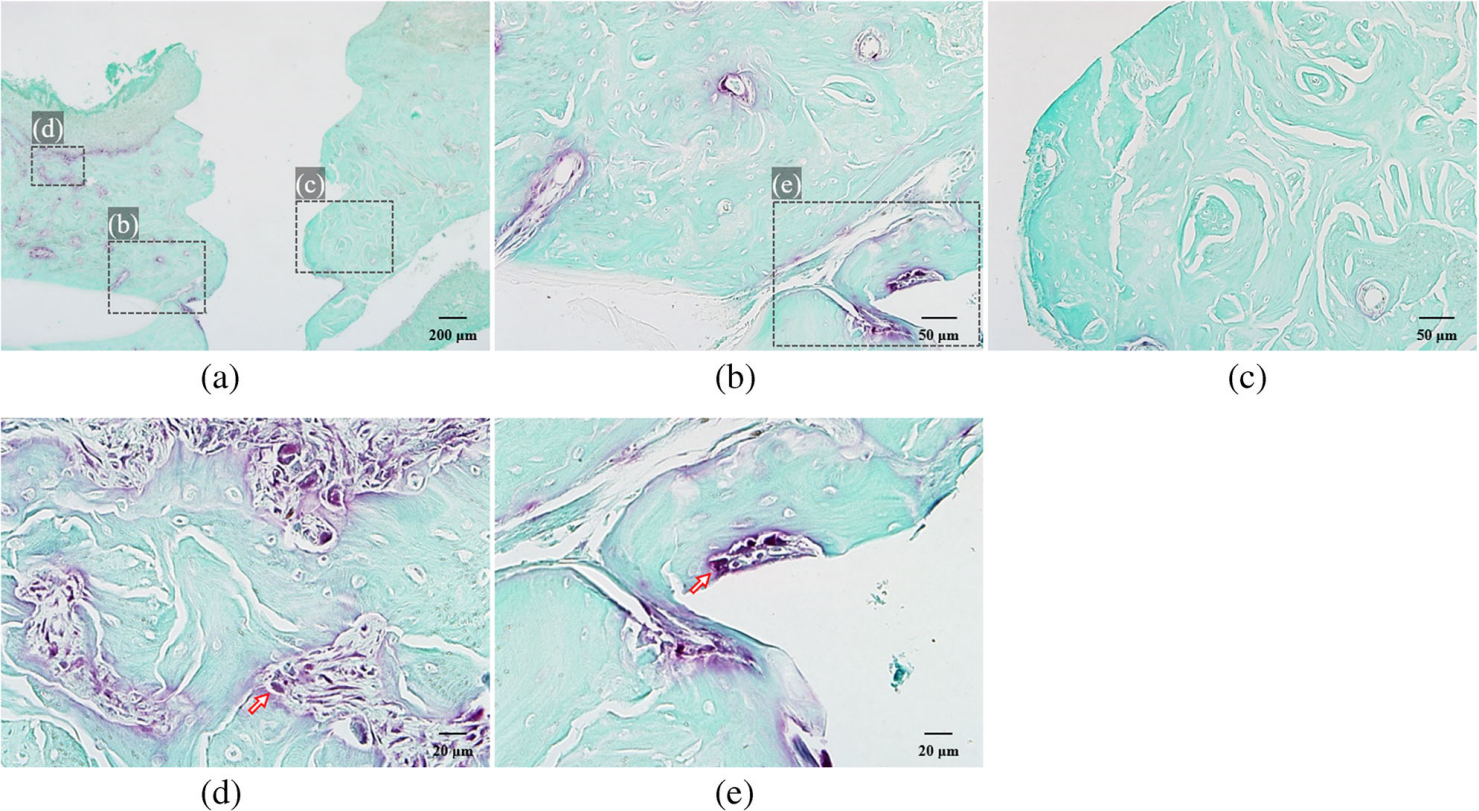
TRAP-stained specimen slide: (a) nt-p65-TMD-treated and silk-induced peri-implantitis group C (magnification ×40), (b) multi-nucleated osteoclasts observed in group Cb, which had buccal implant sulcus without topical nt-p65-TMD application (magnification ×200), and (c) osteoclasts absent in group Cp, which had palatal implant sulcus with topical nt-p65-TMD application (×200). (d), (e) One of the osteoclasts, stained as blue spots, was highlighted with an arrow in group Cb (×400).

TRAP-stained specimen slides of group Cb and Cp are shown (Fig. 5). Many multinucleated osteoclasts, stained blue (TRAP-positive) and seen as strong blue spots, were observed in group Cb whereas osteoclasts were not identified on alveolar bone surface in group Cp. One of the osteoclasts was highlighted with an arrow (Fig. 5 d, e).

### Crestal Bone Level Analysis

**Fig. 6:**
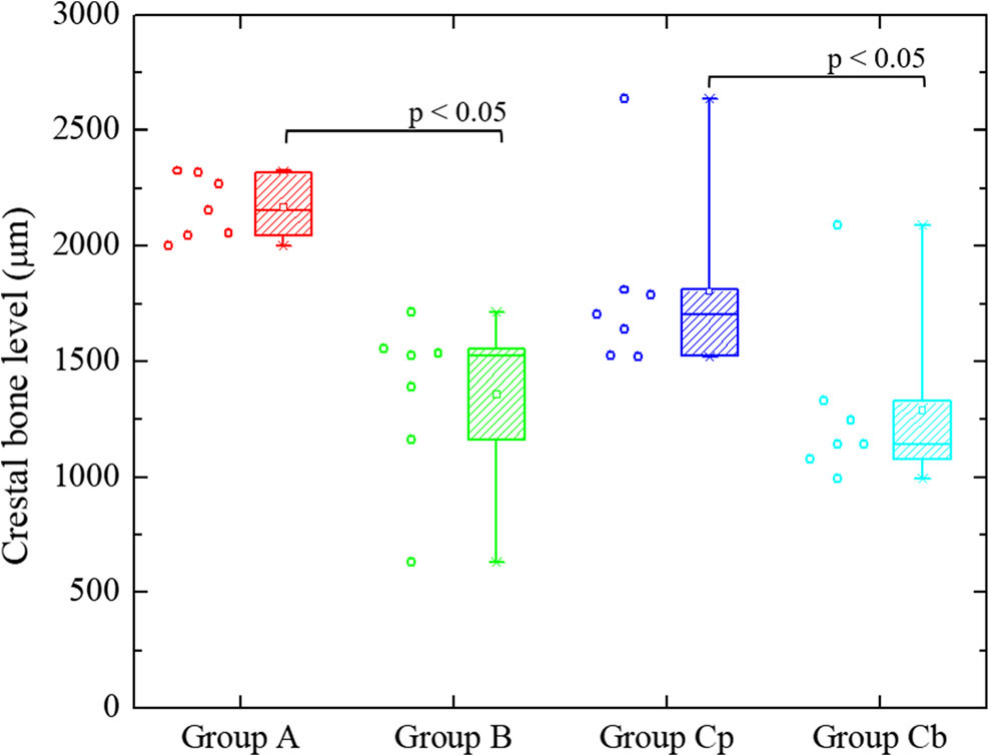
Crestal bone levels of untreated control group A, silk-induced peri-implantitis group B, silk-induced peri-implantitis group Cp, which had palatal implant sulcus with topical nt-p65-TMD application, and silk-induced peri-implantitis group Cb, which had buccal implant sulcus without topical nt-p65-TMD application The crestal bone levels of group A were significantly different from those of group B. The mean crestal bone levels of group A were observed to be higher than those of group B. The crestal bone levels of group Cp were significantly different from those of group Cb. The mean crestal bone levels of group Cp were observed to be higher than those of group Cb.

The crestal bone levels of all groups are presented in Fig. 6. Before the effect of nt-p65-TMD on peri-implantitis was tested, the crestal bone levels of untreated control group A and silk-induced peri-implantitis group B were compared to confirm that silk ligature around the bone screw induced inflammation and bone resorption. The crestal bone levels of group A were significantly different from those of group B at p<0.05 (p=0.00214). The mean crestal bone levels of group A were observed to be higher than those of group B. nt-p65-TMD solution was then applied to the palatal sulcus of silk-induced peri-implantitis group (Cp) while the buccal sulcus of silk-induced peri-implantitis group (Cb) were not treated with the solution. The crestal bone levels of group Cp were significantly different from those of group Cb at p<0.05 (p=0.0214). Thus, it seems that nt-P65-TMD may have contributed to inhibition of inflammatory response and alveolar bone loss.

### Immunohistological Findings on IL-6

**Fig. 7:**
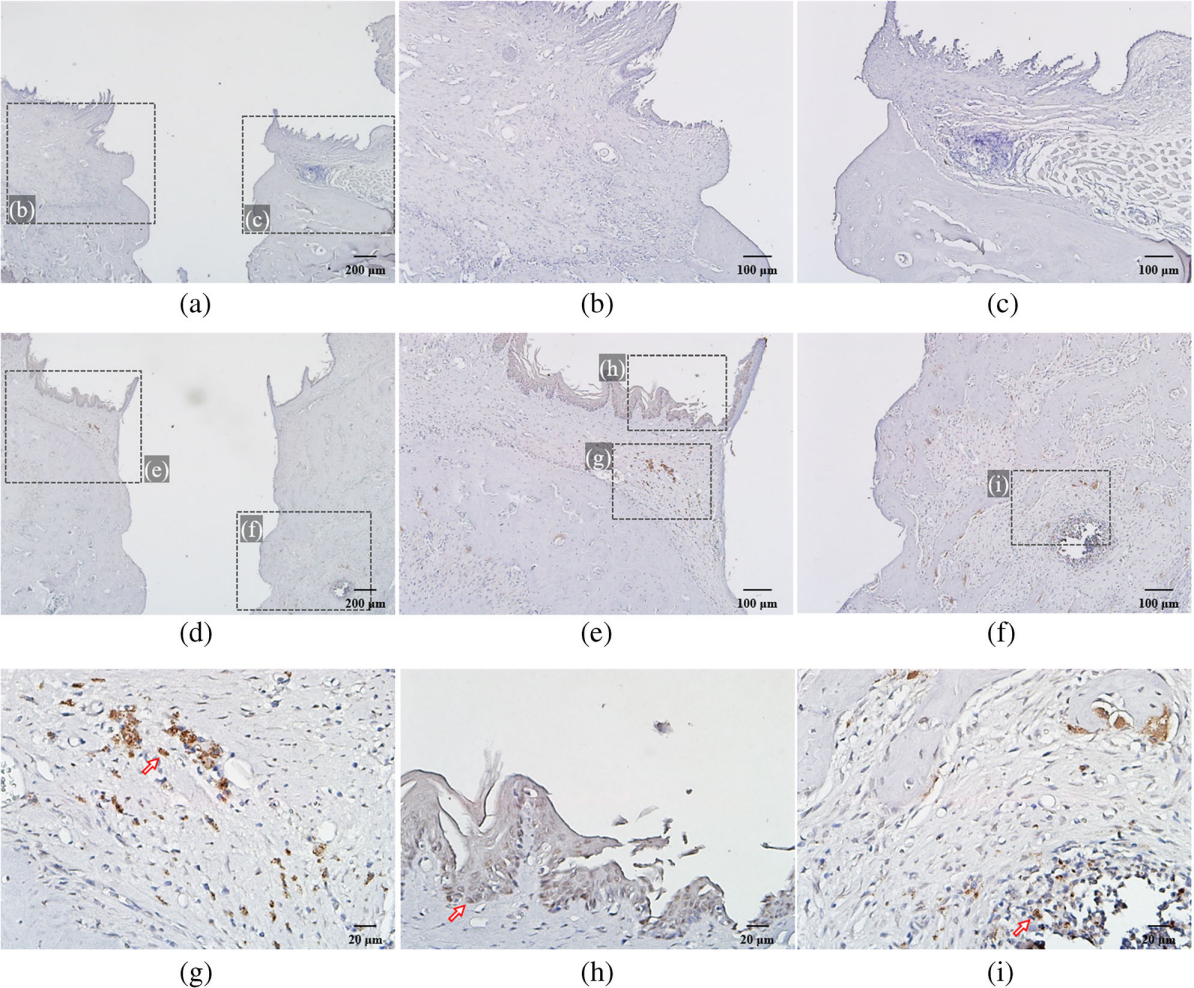
Immunohistological staining: (a) untreated group A (magnification ×40) and (b), (c) group A showed no IL-6-positive cells (×100). (d) Silk-induced peri-implantitis group B (×40). (e), (f) Group B (×100). Group B showed numerous IL-6-positive cells (stained brown) in junctional epithelium (h) and connective tissue (g), (i) while group A showed none (×400).

**Fig. 8:**
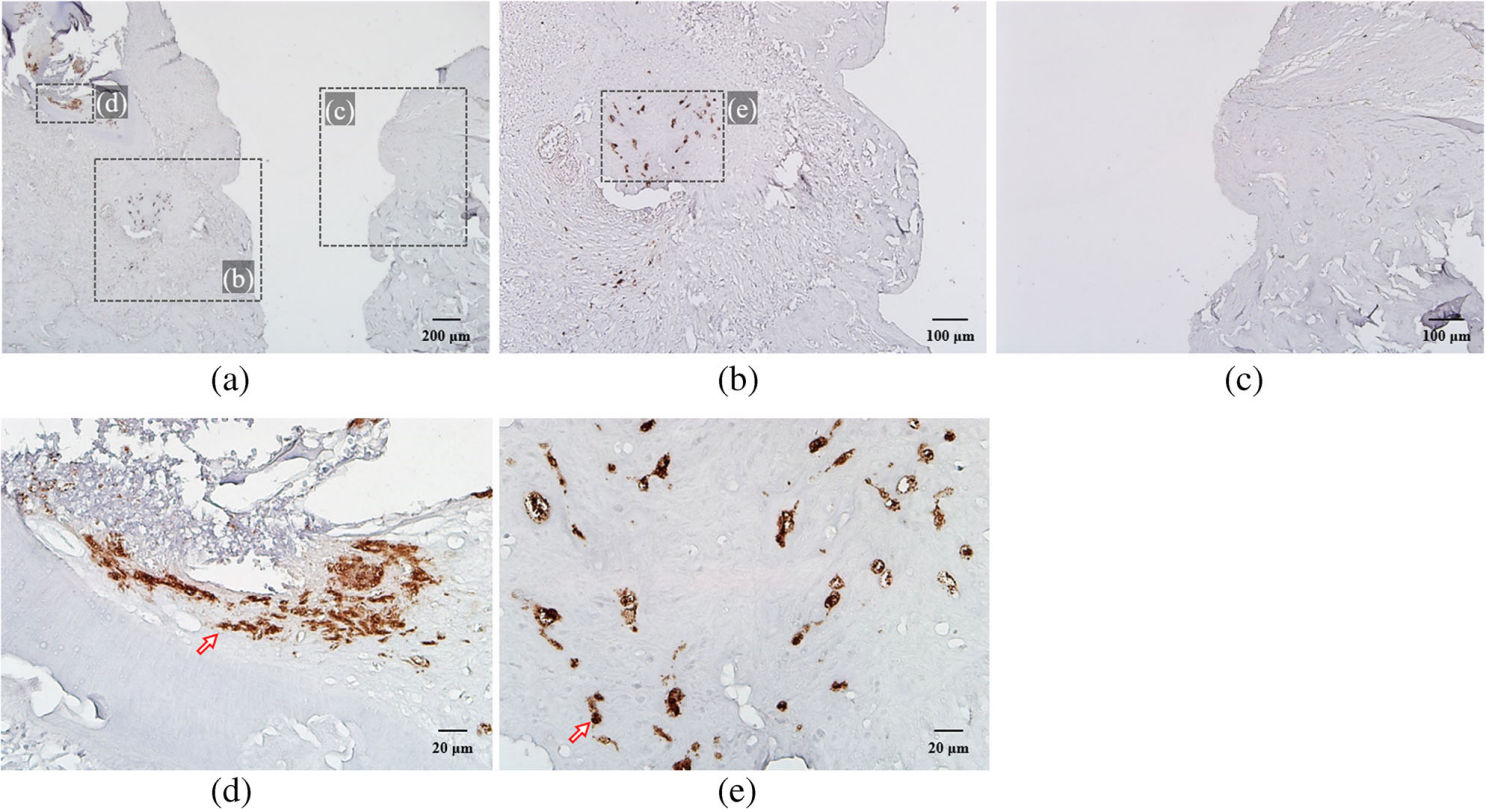
Immunohistological staining: (a) nt-p65-TMD-treated and silk-induced peri-implantitis group C (magnification ×40), (b) group Cb, which had buccal implant sulcus without topical nt-p65-TMD application (×100), and (c) group Cp, which had palatal implant sulcus with topical nt-p65-TMD application (×100). Group Cb showed many IL-6-positive cells (stained brown) in JE (d) and connective tissue (e) while Cp had none (×400).

Immunohistological staining revealed IL-6 expression. The junctional epithelium attached to the implant surface and adjacent connective tissue of group B showed many IL-6-positive cells in contrast to those of group A (Fig. 7). One of the IL-6-positive cells, stained brown, was highlighted with an arrow in JE (Fig. 7 h) and connective tissues (Fig. 7 g, i) of group B. As for group C, group Cb showed many positive cells in JE and surrounding connective tissue while group Cp had none (Fig. 8). One of the IL-6-positive cells, stained brown, was highlighted with an arrow in JE (Fig. 8 d) and connective tissues (Fig. 8 e) of group Cb. It is highly likely that inhibition of IL-6 expression in group Cp was caused by inhibition of NF-kB transcriptional activity by nt-p65-TMD.

## DISCUSSION

The results of this study showed increased bone resorption, apical migration of junctional epithelium, and infiltration of inflammatory cells and osteoclasts around the peri-implantitis sites. These results are consistent with other studies that placed ligatures around the implants and induced peri-implantitis [10, 11].

NF-kB complex activates transcription of many pro-inflammatory proteins, one of them being IL-6 [12]. Park *et al.* reported that transduction of nt-p65-TMD by PTD effectively and consistently suppressed NF-kB functions, reducing the secretion of pro-inflammatory cytokines such as TNF-α, IL-1β, or IL-6 [6]. Other studies have shown the crucial role of NF-kB in bone homeostasis by combined deletion of NF-kB subunits. The knock-out mice lacked osteoclasts and thus showed severe osteopetrosis [13, 14]. Another study showed that NF-kB subunits induced osteoclast precursor differentiation into osteoclasts, confirming that osteoclast formation and activity require NF-kB [15] [6]. The effects of NF-kB inhibition specifically in peri-implantitis were reported in He Cy *et al.* The study used a canine model of ligature-induced peri-implantitis and inhibited NF-kB with pyrrolidine dithiocarbamate (PDTC). The suppression of NF-kB reduced TLR4 protein expression, IL-1, IL-6, IL-8, and TNF-α production, periodontal ligament fibroblasts (PDLFs) apoptosis and induced PDLF proliferation [16].

Since NF-kB plays a key role in inducing inflammatory responses, NF-kB functions were targeted for suppression using nt-p65-TMD to reduce inflammation and bone resorption at peri-implantitis sites in this study. Histopathological and crestal bone level analyses of group C showed decreased apical migration of JE and less bone resorption, as well as fewer inflammatory cells and osteoclasts in group Cp relative to group Cb. However, in contrast to He CY *et al.*, which used systemic application of PDTC via intraperitoneal injection, this study used PTD to deliver p65-TMD to cells in peri-implant sites by needle-free topical administration of nt-p65-TMD on gingival sulcus around implants.

Since their identification about 25 years ago, PTDs have been extensively researched as vectors for gene therapy. Consisting of 6–30 amino acid long synthetic, or naturally occurring peptides, they can efficiently deliver a range of materials including peptides, proteins, nucleic acids, liposomes, nanoparticles, viral particles, radioisotopes, and fluorescent probes into cells. They can also deliver therapeutic agents directly and even target certain cell types if designed, leading to less cellular toxicity [17]. In this study, engineering cell-specific PTD is not necessary to reduce cellular toxicity because peri-implant sites in the oral cavity are easily accessible. In many cases, cell-specific PTDs have to be developed to avoid issues associated with non-cell-specific PTDs. Non-cell-specific PTDs can cause off-target side effects by non-specific cellular uptake and they also need to be administered in high concentration to achieve adequate levels in treatment area. Intra-oral, topical delivery can limit the transduction activity of non-cell-specific PTDs to peri-implant sites because target cells are accessible with limited diffusion [17]. Thus, the side effects from non-cell-specific PTDs can be circumvented for peri-implantitis treatment. Since PTD has great potential for clinical use in dental treatment, this study opted for PTD as a carrier of p65-TMD to suppress the NF-kB signaling pathway, which causes an inflammatory response in hosts. Based on the results, suppression of NF-kB functions by p65-TMD-linked PTD seemed safe and successful in rats. Needle-free topical application of therapeutic agents via PTD used in this study can benefit patients in a clinical setting since it is pain free and has fewer side effects.

Since this study, with its minimal animal sample size, seems to show promising results regarding NF-kB complex inhibition in controlling inflammation, a follow-up study with a larger sample size should investigate nt-p65-TMD-linked PTD application with respect to a range of concentrations, intervals, and frequencies. Molecular mechanisms must be elucidated before applying nt-p65-TMD in gene therapy for patients with peri-implantitis in the future.

## CONCLUSION

According to the results of this study, it is clearly observed that nt-p65-TMD-linked PTD inhibited NF-kB functions and reduced inflammation and bone resorption at peri-implantitis sites in rats. Its use should be further explored and tested because successful local inhibition of the NF-kB complex pathway may help control inflammation and bone resorption in patients with peri-implantitis. In conjunction with gene therapy, our topical medication approach with its reduced systemic side effects may serve as a powerful tool to treat patients in the future.

## Data Availability

The datasets generated during and/or analyzed during the current study are available from the corresponding author on reasonable request.

## Code Availability

There is no code available.
